# Spectral-Domain Optical Coherence Tomography for Macular Edema

**DOI:** 10.1155/2014/191847

**Published:** 2014-05-14

**Authors:** Emmerson Badaró, Eduardo Novais, Larissa Maria Prodocimo, Juliana M. Ferraz Sallum

**Affiliations:** Department of Ophthalmology, Federal University of São Paulo, 821 Botucatu Street, 1st Floor, Vila Clementino, 06023-062 São Paulo, SP, Brazil

## Abstract

Optical coherence tomography (OCT) is a rapid noncontact method that allows in vivo imaging of the retina and it has become an important component in clinical practice. OCT is a useful ancillary tool for assessing retinal diseases because of its ability to provide cross-sectional retinal images and quantitatively analyze retinal morphology. The introduction of spectral-domain OCT provided major improvements in image acquisition speed and image resolution. Future studies will address how these major technologic advances will impact the use of OCT in research and clinical practice.

## 1. Introduction

**Figure 1:**
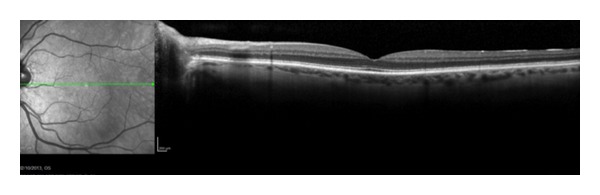
A spectral domain optical coherence tomography line scan of a normal eye.

Optical coherence tomography (OCT) was introduced in 1991 as a noninvasive in vivo ophthalmic imaging technique for facilitating retinal thickness measurement [1, 2]. This high-resolution, cross-sectional imaging technique allows detailed assessment of retinal thickness and morphologic evaluation of the neurosensory retinal layers, retinal pigment epithelium (RPE), and choroid (Figure 1).

OCT is an interferometric imaging technique that generates cross-sectional images by mapping the depth-wise reflections of low-coherence laser light from different kinds of tissue. Spectral-domain OCT (SD-OCT) or Fourier-domain OCT refers to Fourier transformation of the optical spectrum of the low-coherence interferometer. The optical spectrum output of an interferometer exhibits peaks and troughs, and the period of such modulation is proportional to the optical path differences in the interferometer. Imaging of multilayer objects, such as the retina, results in various modulation periodicities representing the depth of each layer. SD-OCT identifies the retinal thickness from the RPE to the inner limiting membrane [3].

The most important advantage of SD-OCT compared with conventional time-domain OCT (TD-OCT) technique is the increased scanning speed [2, 4, 5]. With SD-OCT imaging, acquisition of 25,000 to 100,000 scans/second is routinely possible [5]. This is more than 100 times faster than the TD technique. The axial image resolution of OCT depends on the bandwidth of the low-coherence light source. Most OCT systems use superluminescent diodes with a bandwidth of about 20 to 50 nanometers (nm) that allows axial resolution of 5 to 10 microns. Commercial OCT systems use light sources between 800 and 900 nm wavelengths, allowing for good retinal imaging. SD-OCT has improved visualization of the intraretinal morphologic features, which facilitates evaluation of the integrity of each retinal layer. The image quality of all SD-OCT instruments is sufficient to delineate as many as 10 retinal layers [4]. Another important advantage of SD-OCT instrumentation is the possibility to obtain three-dimensional scans allowing for visualization of structural changes in the vitreoretinal interface and the retina in large areas [4].

This report is an evidence-based review of the increasing role of OCT in the diagnosis and management of ocular disorders, particularly in age-related macular degeneration (AMD), diabetic macular edema (DME), and retinal vein occlusion (RVO).

## 2. Macular Edema

Macular edema, whether associated with diabetic retinopathy, uveitis, retinal vascular diseases (branch and central retinal vein occlusions (BRVO/CRVO)), or postcataract (Irvine-Gass) macular edema, or in idiopathic cases, can lead to severe visual loss if undetected and untreated. Current diagnostic techniques for assessing macular edema, including biomicroscopy, fundus photography, and fluorescein angiography (FA), are widely used, but interpretation of the results can be subjective, and subtle changes in retinal thickness in early stage macular edema may not be evident using these techniques.

OCT is considered the best reference standard for detecting and quantifying macular edema compared to ultrasound, retinal thickness analysis, and scanning laser ophthalmoscopy [6, 7]. Compared to biomicroscopy and FA, OCT has superior sensitivity and greater resolution for detecting macular edema and subretinal fluid [8–10].

**Figure 2:**
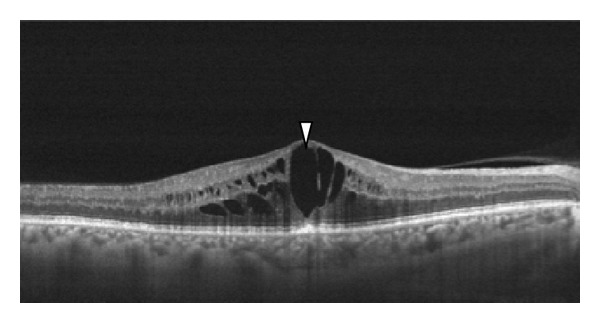
Cystoid macular edema can be seen clearly on OCT scans as multiple circular cystic spaces in the retina, indicating intraretinal edema (white arrowhead). The cystic spaces are round and originate around the outer plexiform layer.

Cystoid macular edema (Figure 2) can be seen clearly on OCT scans as multiple circular cystic spaces in the retina, indicating intraretinal edema. The cystic spaces are round and originate around the outer plexiform layer but can progress to involve the photoreceptor layer and the inner retinal layers. Occasionally, cystic retinal edema can enlarge CRT and have the appearance of a foveal pseudocyst.

### 2.1. OCT in Diabetic Macular Edema

 DME is the most common cause of moderate visual loss in patients with diabetes [11]. In 1998, Hee et al. were the first to use OCT to measure the retinal thickness in patients with DME [12]. As a result, OCT imaging has rapidly been integrated into diagnosis and management of DME in routine clinical practice and clinical trials [3]. OCT was found to be highly sensitive and specific for detecting DME compared to other diagnostic modalities such as FA and the Retinal Thickness Analyzer (Talia Technology Ltd., Neve-Ilan, Israel) [7, 13].

**Figure 3:**
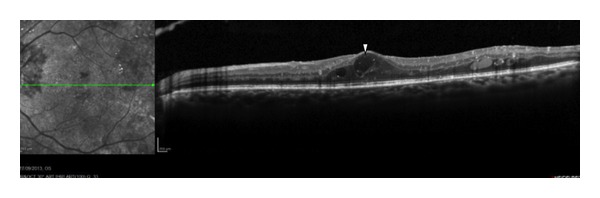
An OCT horizontal line scan of a 62-year-old man with diabetic retinopathy and macular edema-intraretinal cysts (white arrowhead).

The prevalence of DME increases from 0% to 3% in individuals with recent diagnoses of diabetes to 28% to 29% in those with diabetes for longer than 20 years [14], making it the principal mechanism of visual loss in patients with nonproliferative diabetic retinopathy [15]. The pathogenesis is not understood completely, but intraretinal fluid develops secondary to microaneurysm formation, increased vascular permeability, and breakdown of the blood-retinal barrier [5, 16] (Figure 3). Vascular endothelial growth factor (VEGF) may play an important role [17]. The ability to detect and quantify the central retinal thickness in patients with clinically diagnosed DME is important when treating patients with diabetes. Other causes of limited visual function include macular ischemia [18], photoreceptor dysfunction [19], and accumulated subfoveal hard exudates [20].

DME had been characterized as focal or diffuse based on clinical examination and FA findings [11]. OCT allows for more precise evaluation of the retinal pathology in DME, including the retinal thickness and edema, vitreomacular interface abnormalities, subretinal fluid, and foveal microstructural changes. Additional advantages include the possibility of correlations with FA and the ability to quantitatively monitor responses to treatment of DME by laser, intravitreal pharmacotherapies, and vitreoretinal surgery [5]. The test-retest variability of OCT measurements for retinal thickness is less than 10% in patients with diabetes both with and without DME [21, 22]. Therefore, a change in OCT thickness exceeding 10% is often considered clinically relevant, and the change in relative central subfield thickening is minimal, with a mean decrease of only 6% between 8 a.m. and 4 p.m. [23]. Before OCT technology, precise monitoring of the CRT was impossible.

**Figure 4:**
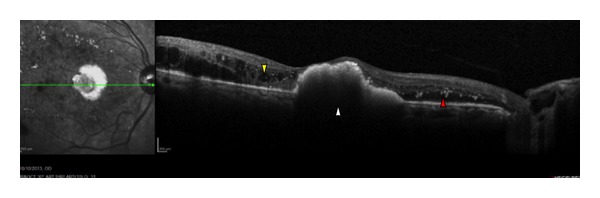
An OCT horizontal line scan of a woman with diabetes, with a juxtafoveal accumulation of hard exudates (white arrowhead) and substantial fluid at the level of outer plexiform layer (yellow arrowhead). Diffuse hyperreflective hard exsudates can also be seen (red arrowhead).

The OCT findings were well correlated with other evaluation techniques for DME. Although there is a moderate correlation between the retinal thickness measured by OCT and visual acuity (VA), OCT cannot replace the VA because there is a high degree of variability [3]. Macular edema and thickness are only two factors among many that affect the VA in patients with DME. Macular ischemia and foveal exudates (Figure 4) also contribute to the poor prognosis. Recently, log changes in OCT have been proposed as a better method of analyzing OCT parameters instead of using absolute values, since the same degree of absolute changes in microns may be qualitatively different depending on the baseline retinal thickness [24].

To investigate the relationship between VA and the CRT measured by OCT, 251 eyes of 210 patients with DME were enrolled in a cross-sectional and longitudinal randomized clinical trial [25]. The Diabetic Retinopathy Clinical Research Network documented a modest correlation between the best-corrected VA and the CRT measured by OCT before focal laser photocoagulation and a modest correlation between changes in VA and changes in thickening of the center point measured by OCT during the first year after laser treatment. Many eyes with a thickened macula had excellent VA and many eyes with a macula of normal thickness had decreased VA. The results suggested that OCT measurements, although an important clinical tool, are not an ideal surrogate for VA as a primary outcome in studies of DME.

OCT also allows analysis of the integrity of the outer retinal layers in DME. Various studies have reported that the integrity of the outer retinal layer is linked directly to the visual prognosis [26–29]. Disruption of the hyperreflective photoreceptor inner segment/outer segment junction on OCT, located just above the RPE, may reveal damage to the macular photoreceptors [5].

Many treatment options exist for treating DME, such as focal therapies laser [11], pharmacotherapies, and systemic adjuvant therapies to control glucose levels [30, 31] and blood pressure [32, 33].

In 1985, the Early Treatment Diabetic Retinopathy Study (ETDRS) defined clinically relevant macular edema. The study further reported that focal or grid photocoagulation of eyes with clinically relevant macular edema reduced the 3-year risk of losing three or more lines of VA by 50%, from 30% in the control group to 15% in the laser group [11].

Treating DME with peribulbar triamcinolone acetonide did not significantly improve the central subfield macular thickness measured by OCT or VA [34]. Intravitreal triamcinolone had short-term benefits; however, when triamcinolone was compared with laser in a randomized trial, steroids were inferior at 2 years, with substantially higher rates of complications, surgical interventions, and a three-line visual loss [35].

The newest frontier in treating DME involves the use of anti-VEGF agents. Ranibizumab (Lucentis, Genentech, Inc., South San Francisco, CA), a monoclonal antibody fragment with binding affinity for VEGF-A, has been evaluated for treating DME [36]. Pilot studies found that intravitreal ranibizumab reduced macular edema on SD-OCT and improved VA in patients with DME [17]. Ranibizumab received Food and Drug Administration approval for treating DME based on the results of major clinical trials [37]. The benefits of ranibizumab for improving the VA and central foveal thickness on SD-OCT can be observed as early as 7 days after treatment initiation; however, whether and for how long the beneficial effects of ranibizumab on retinopathy severity and progression persist after therapy cessation also need to be determined [37]. Diabetic Retinopathy Clinical Research Network is also evaluating this question [38].

### 2.2. OCT in Age-Related Macular Degeneration

**Figure 5:**
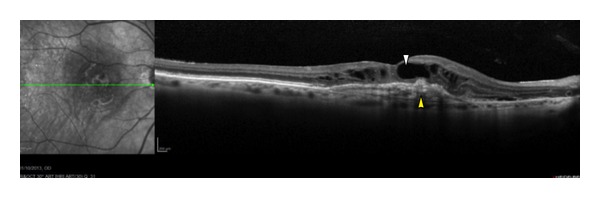
A line scan of an eye with macular edema secondary to an active exudative AMD. The technique allows for visualization of the cystic spaces (white arrowhead) and other changes in the retinal layers. Note the hyperreflective layer underneath the neurosensory retina suggestive of the neovascular membrane complex (yellow arrowhead).

SD-OCT is becoming an integral component in the diagnosis and management of AMD. OCT testing provides qualitative and quantitative assessment of various AMD presentations and detects early nonexudative AMD changes, such as drusen and RPE atrophy, and exudative AMD findings, such as intraretinal fluid, RPE detachment, retinal angiomatous proliferation, and choroidal neovascular membranes (Figure 5). OCT may be used to measure changes in drusen volume and to measure geographic atrophy incensement [39]. RPE tears are not infrequent among eyes treated with intravitreal anti-VEGFs, and the presence, increased height, and shorter duration of PED are potential risk factors for RPE tears associated with anti-VEGF therapy. OCT gives us an important tool in detecting and measuring both PED increase and RPE tears [40].

**Figure 6:**
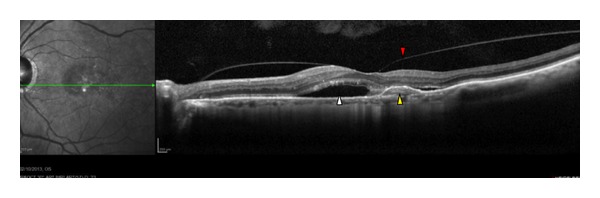
An OCT line scan of a 73-year-old man with exudative AMD. The white arrowhead shows that the hyporeflective space below the neurosensory retina is clearly visible, suggesting the presence of fluid. Yellow arrowhead represents a hemorrhagic detachment of the retinal pigment epithelium (PED) and vitreomacular traction in addition to vitreous alterations (red arrowhead).

The ability to detect intraretinal fluid on OCT images is an effective way to guide treatment and retreatment, because intraretinal fluid is associated with active neovascular membranes. OCT imaging features that may be associated with choroidal neovascularization include thickening or fragmentation of the RPE, choriocapillaris, intraretinal and subretinal fluid accumulation related to neovascularization exudation, and pigment epithelial detachment [41] (Figure 6). Serial SD-OCT scanning is essential to monitor the responses to anti-VEGF therapies, which decrease the activity of the neovascular membranes, with a resultant reduction in intraretinal edema and improved retinal architecture.

### 2.3. OCT in Retinal Vein Occlusion

RVO is a common, sight-threatening retinal vascular disorder and the second most common retinal vascular disease after diabetic retinopathy. The clinical characteristics, prognosis, and response to treatment are affected by the location of the occlusion in the retinal venous vasculature, the presence of macular edema, and the extent of the retinal nonperfusion. The location of the occlusion may affect the severity of the clinical manifestations.

**Figure 7:**
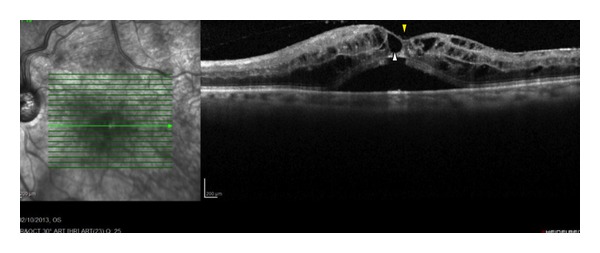
Macular edema in a 75-year-old woman with CRVO. There are several cystic spaces in the retinal layers (white arrowhead), although the foveal depression is preserved (yellow arrowhead).

Macular edema, a major cause of visual loss in patients with RVO [42], involves a spectrum of different pathologic retinal changes, including intraretinal fluid accumulation with diffuse retinal thickening or formation of cystoid spaces, subretinal fluid accumulation, or macular traction due to epiretinal membrane formation (Figure 7). SD-OCT assessment of the VA and measurement of retinal thickness and structural changes provides useful information for determining treatment strategy for RVO.

Some parameters seen on SD-OCT seem to be correlated negatively with visual recovery after RVO, such as foveal thickness, serous retinal detachment, central cystoid spaces, and pigment epithelial changes. When a very thick fovea (more than 700 microns) is seen on SD-OCT, an ischemic form of CRVO should be suspected [43]. Loss of the foveal photoreceptor junction line and absence of the inner retinal layers on late-stage SD-OCT images are correlated with poor visual outcomes in patients with CRVO [44] and BRVO [45–47]. In addition, loss of the inner retinal layers on late-stage SD-OCT images is correlated significantly with macular ischemia diagnosed in early FA studies [48]. These specific structural changes suggest that SD-OCT can be an important tool for evaluating and managing macular edema and predicting the long-term visual prognosis in patients with CRVO. The relationship between the CRT and VA has not been well established in CRVO [48].

The current management of macular edema secondary to CRVO relies on two different approaches involving intravitreal therapy: anti-VEGF therapy [49, 50] or steroids [51]. Management of BRVO includes grid laser photocoagulation, steroids, or anti-VEGF agents.

## 3. Conclusion

SD-OCT technology has revolutionized the evaluation and treatment of macular edema. The technology facilitates quantitative assessment of the degree of retinal thickness, which has proved to be useful in the diagnosis, management, and follow-up of patients with macular edema, including assessment of foveal microstructural changes. OCT is a highly accurate and reproducible method for diagnosing macular edema that compares well to the standard clinical examination, ETDRS photos, and FA. Although it is useful for anatomic study, OCT is not an effective surrogate for functional tests such as VA measurement in the assessment of macular edema [3]. Future research in SD-OCT imaging likely will result in improvements in resolution, speed, image registration, three-dimensional imaging, and the ability to combine SD-OCT with other various diagnostic modalities that will further our ability to evaluate macular edema in clinical practice and trials for our patients with diabetes, RVO, and exudative AMD. Many therapeutic modalities exist for treating those conditions, with some believed to be an exciting new frontier.
